# Adaptation of COMPASS for use in Autism-Specific Schools in Australia: A Cluster Randomized Trial

**DOI:** 10.1007/s10803-024-06345-y

**Published:** 2024-06-01

**Authors:** Abigail M. A. Love, Ru Ying Cai, Jennifer Stephenson, Emma Gallagher, Michael D. Toland, Vicki Gibbs

**Affiliations:** 1 Autism Spectrum Australia, Aspect Research for Autism Practice, Chatswood, NSW 2067 Australia; 2 School of Education, Faculty of Arts, Macquarie University, Sydney, NSW 2109 Australia; 3 University of Toledo, The Herb Innovation Center, Toledo, OH 43606 USA; 4 FMH Translational Research Collective, The Faculty of Medicine and Health, The University of Sydney, Sydney, NSW 2006 Australia

**Keywords:** Autism, COMPASS, Education, Goal attainment scaling, Individualized planning

## Abstract

Autistic students are educated in Australia across a variety of contexts and almost all educators use individualized goal-setting as a way of ensuring appropriate accommodations and curriculum modifications. Educators experience similar challenges when developing individualized goals for students, including lack of a standardized process for goal setting, inconsistent support networks, and challenges with data-driven goal-setting. The purpose of our research was to adapt and trial the Collaborative Model for Competence and Success (COMPASS), a research-based intervention aimed at improving the individualized goal-setting process for autistic students. Our primary hypothesis was that autistic students who participate in COMPASS would demonstrate more growth on their individualized outcomes relative to autistic students who receive services as usual (SAU). To answer our primary research question, we applied a single-blind cluster randomized trial. Participants were randomized into one of two groups: (a) a COMPASS intervention group and (b) a SAU group. Results indicate that students whose teachers received the intervention made more progress on their final individualized goals than those who received SAU, replicating previous findings regarding COMPASS in the US. Also, the adaptation of COMPASS for an Australian context showed high rates of satisfaction and fidelity. The success of this intervention in improving the progress that students make on their individualized goals demonstrates the benefits of a standardized intervention that supports teachers and families in this critical practice.

## Introduction

In Australia, studies examining the prevalence of autism among children have yielded rates between 1 in 22 and 1 in 70 (May et al., 2017, 2020; Nielsen et al., 2024; Randall et al., 2016). Variations in the prevalence rates fluctuate due to diverse methodologies and data sources (e.g., education data, medical records, population data), but findings are consistent in concluding a rising prevalence rate due to many factors including improved diagnoses and funding models for early intervention services (Nielsen et al., 2024). Teaching students on the autism spectrum requires awareness of how autism can impact learning and development and the range of curriculum adjustments that are available to assist autistic students. Autistic students learn best when provided with an individualized educational program that recognizes their unique strengths and includes adjustments and additional supports to ameliorate any social, communication, or learning difficulties (Schuck et al., 2022). The use of individualized programs also helps to promote independence in educational and social activities by addressing language, social, and adaptive skills that are not explicit parts of a traditional curriculum (Boavida et al., 2010; Müller et al., 2022). In the later years of schooling, goals can also be designed to support the transition to adulthood, with a focus on vocational and residential independence and written to align with transition planning that happens before students graduate from school (Hagner et al., 2014; Lee & Kim, 2021; Wagner et al., 2012).

Individualized strategies and supports are key characteristics of effective school programs for students on the autism spectrum (Roberts & Webster, 2022). Individualized programs are a fundamental practice to document person-centered goals and accompanying strategies that help students succeed in their educational environments (Boavida et al., 2010; Christle & Yell, 2010; Stephenson & Carter, 2015). For our research, the term “Individualized Plan” (IP) will be used to refer to a student’s individualized educational plan, sometimes called an Individual Educational Plan (IEP) or an Individual Learning Plan (ILP). In Australia (unlike the US), there are no prescribed standards or processes for developing IPs. Researchers have found inconsistencies in the quality of IP goals; yet, they have begun to show that high-quality IP goals (e.g., those that are measurable and student-focused) lead to better student outcomes (Findley et al., 2022; Landmark & Zhang, 2013; Ruble & McGrew, 2013; Sanches-Ferreira et al., 2013).

The heterogeneity of students with disability, specifically those on the autism spectrum, presents a challenge in creating high-quality, meaningful goals that can be tracked using data-driven strategies (Boavida et al., 2010; Carter et al., 2022; Findley et al., 2022; Ruble et al., 2021). Studies assessing the quality of IP goals have found they tend to be too broad, lack functionality, do not address skills within natural contexts, and are unmeasurable (Boavida et al., 2010). Moreover, despite US mandates requiring parent input in individualized planning, parents often report that they are left out of the decision-making process (Ruppar & Gaffney, 2011) and need to fight for adequate services over long periods (Kurth et al., 2020). According to the Australian educational needs analysis (Saggers et al., 2015), parents and caregivers of autistic students reported a range of experiences with family involvement from very positive contributions to a lack of family involvement, as well as experiences with schools actively discouraging family involvement. Educators continue to report barriers to creating quality IP goals for students, including a lack of standardized processes for goal setting, incomplete avenues for support, and challenges with data-driven goal-setting (Carter et al., 2022; Müller et al., 2022). A standardized intervention designed to collaboratively problem-solve and support teachers, families, students, and other stakeholders to develop meaningful IP goals along with associated teaching plans is a way to reduce burdens associated with goal-setting and increase student growth and progress on critical IP goals.

### COMPASS

The Collaborative Model for Competence and Success (COMPASS; Ruble et al., 2012a, 2023) is a manualized research-based intervention guiding collaboration within a team that supports a student during the IP goal-setting process. Team members include an autism-specific consultant, the student’s classroom teacher, the caregivers, and the student. Team members come together several times during the academic year for two main components of the COMPASS intervention: (a) a caregiver-teacher consultation that results in individualized goals and teaching plans and (b) four coaching sessions where the consultant and teacher collaborate, problem-solve, and measure student progress to maximize student progress towards their IP goals. Students are encouraged and supported to participate in the initial consultation as well as the coaching sessions.

The accumulation of evidence for the effectiveness of COMPASS has been steadily increasing and includes a range of studies since 2010. The first published study (Ruble et al., 2010) demonstrated that students made more progress on their IP goals than students who did not receive COMPASS or any intervention from the research team. Next, COMPASS was tested using face-to-face coaching compared to web-based (video-conference) coaching (Ruble et al., 2013), and results showed the intervention improved students’ progress on their IP goals, regardless of the mode of delivery. A third research study focused on students who were preparing to leave secondary school, with results indicating that COMPASS was effective among older students (Ruble et al., 2018b). Further, Wong et al. (2017) demonstrated that the impact of COMPASS on student outcomes was mediated by teaching quality and student engagement. Across all three studies (Ruble et al., 2010; 2013; Ruble et al., 2018b), COMPASS was delivered with high fidelity by researchers, and student IP outcomes were better for those who participated in the intervention.

### Community-Setting

As a next step to supporting the use of COMPASS in schools by practitioners, COMPASS researchers explored implementation by school- or community-based consultants rather than members of a research team (i.e., a train-the-trainer model). As such, a training package for community-based consultants was developed by Ruble et al. (2022) and results demonstrate, as observed in past studies, improved student outcomes and high fidelity of implementation. Subsequently, Ogle and colleagues (2023) studied the impact of varying the type of feedback (face-to-face vs. email) and dosage (one, two, or four coaching sessions) of teacher coaching feedback to determine whether all four of the standardized coaching sessions were needed to achieve consistent student outcomes. Although the original design was impacted by COVID-19, results show that *type* of feedback did not affect student outcomes. Yet, the *dosage* was an active mechanism, as receiving no coaching sessions or only one coaching session did not significantly change student outcomes whereas receiving two or four sessions changed student outcomes. COMPASS has also been shown to influence teacher outcomes, such as increased teacher self-efficacy (Love et al., 2020). It is important to note that all COMPASS research through 2022 has been conducted in US mainstream schools, where students on the autism spectrum are educated with the support of a Special Education teacher in their least restrictive environment (Individuals with Disabilities Education Improvement Act, 2004). It is unknown whether the positive effects of COMPASS can be demonstrated in contexts outside of the US mainstream environment. Additionally, the training package has only been used by *external consultants* (i.e., professionals who are autism specialists from local, regional, state, or out-of-state areas to work in a group of schools or have a caseload of students on the autism spectrum). External consultants were trained in COMPASS and then delivered the intervention with their teachers and families that were on their caseload. It is unknown how the effects of the training package for COMPASS would unfold if *internal consultants* are used (i.e., professionals employed by a school to work within a specific school). Both external and internal consultants are used across the United States and Australia and models vary depending on the funding, skills of the consultants, legislation of the state and country, and school makeups. Therefore, understanding the success of COMPASS with both external and internal consultants can help identify the most appropriate users of COMPASS.

## Purpose of Our Study

In Australia, a substantial proportion of autistic children attend specialist settings (ABS, 2016). Our study represents a close collaboration between a research team and a group of autism-specific, independent schools in New South Wales, Australia, which are run by Autism Spectrum Australia, the largest autism-specific service provider in Australia. This research evolved from organic meetings between researchers, teachers, and school consultants who expressed a need for a more standardized and efficient way to set and measure IP goals for their students on the autism spectrum. We were therefore interested in how COMPASS would support consultants and teachers with autism-specific expertise already working at an autism-specific school.

The purpose of our study was to explore the effectiveness of COMPASS adapted for the Australian context on student outcomes. First, we hypothesize autistic students who participate in COMPASS would demonstrate more growth on their individualized outcomes (i.e., at midterm and final assessment) relative to autistic students who receive services as usual (SAU). Second, we hypothesize that COMPASS teachers will have higher adherence compared to teachers providing SAU. Third, adherence will improve across coaching sessions for COMPASS teachers. Finally, with context-specific adaptations, we hypothesize COMPASS can be implemented in an Australian autism-specific classroom with high levels of satisfaction across stakeholders.

## Method

### Community Involvement Approach

The expectation that the intervention and research teams include input from people with lived experience with autism has become a critical design component of autism research for ensuring validity and alignment with community perspectives (Hollin & Pearce, 2019; Pellicano & den Houting, 2022). Therefore, our research project used the community-based participatory research (CBPR) approach to create knowledge user-research collaborations throughout the research cycle. CBPR is a collaborative research method and an “umbrella term” for approaches aiming to equitably involve community partners in the entire research process (Minkler & Wallerstein, 2003). We aimed to engage users of the module (teachers and consultants) and those with whom the module is ultimately meant to benefit (autistic students and their families) in the design. This research has been co-produced, including two autistic researchers involved in the research design, ethics application, data collection and management, data analysis, and reporting of results. Both autistic researchers hold education qualifications and utilized their teaching, lived experience, and professional knowledge to help guide and shape the research methodologies, data collection instruments, and research outputs to ensure their relevancy to the autism communities. Additionally, our team had sustainability of research findings in mind; that is, to use the results of this study to understand how improved practices around individualized planning could be continued within this group of schools beyond intervention by the research team.

### Research Design

A single-blind cluster randomized trial was applied. Specifically, consultants were randomized into one of two groups: (a) consultants who were trained in COMPASS and delivered the intervention with teachers and families across one school year; (b) consultants who delivered an individualized planning procedure that was considered services as usual (SAU) at the schools in our study.

### Consultants

In the present study, consultants were *internal consultants* (i.e., trained educators employed by Aspect in senior advisory and support positions at the school level). Their roles within Aspect are to support and partner with teachers, parents/caregivers, and other members of the school community to ensure that high-quality, evidence-based practice is delivered.

### COMPASS

COMPASS is delivered through the consultant, who supports the teacher and family in designing and implementing a student’s IP across a school year. Delivery of COMPASS includes two primary parts: an initial consultation meeting and subsequent coaching sessions. The initial consultation meeting was attended by the student’s teacher, caregiver, and consultant. Additional members attended when possible, including therapy staff or the student themselves. Before the initial meeting, both the caregiver and teachers filled out the COMPASS Profile (Ruble et al., 2023) that gathers information on the student’s personal and environmental strengths and challenges. During the initial consultation, results from the COMPASS Profile are reviewed, focusing on areas of disagreement between home and school or areas of concern. After going through the data, the team engaged in interactive problem-solving and came together to identify three IP goals for the student (communication, social, and learning), with detailed teaching plans developed for each goal. This meeting took up to 3 h but was often (*n* = 11) broken into two 1.5-hour meetings. After these meetings, four coaching sessions (1.5 h each) were planned across the school year. Coaching sessions took place approximately every 8 weeks, spaced out across the four school terms. At least one of the coaching sessions was attended by caregiver(s) and students were invited to attend. However, all coaching sessions were attended by the teacher and consultant to score each student’s individualized goal progress based on evidence (e.g., a video, data). For a complete description of the features of COMPASS, see the COMPASS manual (Ruble et al., 2012a; 2023).

### Services as Usual (SAU)

The comparison group for this study consisted of consultants, teachers, and caregivers who were continuing with the individual planning policy and procedures already set up within Aspect schools. This process included two 30-minute meetings across the school year: an IP meeting at the beginning of the Australian school year (February-March) and a review midyear (September). These meetings were attended by the teacher and the caregiver. Data for the initial meeting came from various assessments, varying across school placements. Each student received one to four individualized goals (depending on their individual needs) across a school year and a teaching plan called an action plan, was written to identify supports and detail the research-based practices and supports that would be in place for the student (see Appendix A). Teachers used a variety of methods to collect data on IP goals including videos, photos, or observational data.

### Adaptations and Modifications of COMPASS

Before we could implement COMPASS, we needed to ensure it was appropriate for the Australian context, as well as the autism-specific classroom. Therefore, to ensure COMPASS would be effective in our community setting, we worked with several stakeholders throughout the entire process to identify areas where the intervention would need to be adjusted. A list of the similarities and differences between the present study and previous COMPASS work is presented in Appendix B. Minor adaptions included language changes to reflect the Australian language, requirements, and spelling and updates to the training package to align with Aspect policies and procedures.

Due to caregiver preference and challenges associated with COVID-19, this study was conducted with a mix of virtual (Zoom) and in-person (face-to-face) sessions. In New South Wales, where the schools in this study were located, a period of predominantly home learning took place between 23rd March and 25th May 2020. Students were able to participate in home learning or attended school on-site for exceptional circumstances; however, there were substantial changes to staffing and programs for those students who attended on-site. Because of these home learning periods and restrictions around travel in Australia, the majority of consultation and coaching meetings were conducted virtually via Zoom. Teams met onsite when possible, but caregiver(s) regularly needed to support their students at home and usually attended meetings virtually. The research team met with the COMPASS consultants in-person for one 8-hour training day in January 2021, but all other interactions were over-the-phone or virtual. Also, due to restrictions and increased home learning, it was important to diversify ways that the COMPASS team could collect IP goal performance data. We used a combination of methods to monitor progress, including videos or pictures submitted by caregiver(s) or teachers, student self-monitoring data, work samples, and staff observational data. We added an observational data document to provide an additional way for teachers to collect data on students’ IP goals and to ensure that caregiver(s), teacher’s assistants, therapists, students, or consultants could make observational notes across settings on students’ IP progress. This modification was needed as well due to many teachers working in co-teaching models and the need to ensure that observational data could come from both teachers.

### Recruitment and Training

This study was conducted in three schools in NSW, Australia. Participants in our study were recruited in a multistep fashion before the 2021 school year, following approval by The University of Sydney Human Research Ethics Committee (Project 2020_761). This study was planned to coincide with Australia’s school year, which matches the calendar year and begins in late January and ends in mid-December. The Australian school year is divided into four terms that run for approximately ten weeks, with two weeks of break in between each term.

Of the nine Aspect schools, three schools indicated their interest and attended a meeting with the research team to hear about our study. After gaining support from each school principal, information about the study was shared with their team of consultants. Interested consultants then met with the research team and provided their consent, before being randomly allocated into either the COMPASS or services-as-usual (SAU) group. We asked consultants to share information about our study with teachers they directly worked with. Interested teachers completed consent and then passed on information for the study to caregivers in their classes. As all students must provide evidence of a formal autism diagnosis to be enrolled at the schools that participated in this study, all students were deemed eligible to participate in this study. Interested caregivers scheduled a phone call with a researcher. If they wanted to participate, they completed a consent form and were enrolled in the study.

Once consent was gathered from all participants, training for COMPASS consultants began before the start of the 2021 Australian school year (January-December). As COMPASS is delivered through the consultant, a COMPASS training package was provided by the original authors of COMPASS and adapted for the Australian context to be delivered to the school consultants (Ruble et al., 2022). The training included two 8-hour training days. See Appendix C for topics covered on both training days. For the SAU consultants, a separate Zoom training was arranged to answer questions about the study and explain data collection expectations. The SAU received no information about COMPASS and no intervention from the research team. They were contacted only at baseline, midterm, and final data time points.

### Participants

Table 1 presents the demographic characteristics of all participants. Consultants (*M*_age_ = 43.8 years, *SD*_age_ = 6.2) had been in a consulting role for an average of 6.9 years (*SD* = 4.4). Consultants worked with between one and four teachers each, depending on their experience in the role and school population size. Teachers (*M*_age_ = 41.0 years, *SD*_age_ = 9.9) each worked with one student and had been teaching for a mean of 15.4 years (*SD* = 9.9). Students (*n* = 36) ranged in age from 5 to 18 years (*M*_age_ = 9.2, *SD*_age_ = 3.2).

**Table 1:** Participant Characteristics by Group

Students (*n* = 36)	SAU		COMPASS	
	*n*	%	*n*	%
Gender				
Male	14	77.8	15	83.3
Female	4	22.2	3	16.7
Ethnicity				
Caucasian	8	44.4	12	66.7
Mixed	1	5.6	5	27.8
Chinese	1	5.6	1	5.6
Bengali	1	5.6	0	0.0
Aboriginal	1	5.6	0	0.0
Filipino	2	11.1	0	0.0
Indian	2	11.1	0	0.0
Missing	2	11.1	0	0.0
Intellectual Disability	9	50.0	8	46.8
Mean Age (years)	9.4 (*SD* = 1.8)		9.0 (*SD* = 4.2)	
SRS-2^1^	94.87 (*SD* = 29.51)		109.45 (*SD* = 23.66)	
**Teachers (*n*** **= 36)**	**SAU**		**COMPASS**	
	*n*	%	*n*	%
Gender				
Male	5	23.8	1	6.7
Female	16	76.2	14	93.3
Ethnicity				
Caucasian	16	76.2	10	66.7
Mixed	1	4.8	0	0.0
Chinese	0	0.0	2	13.3
Bengali	1	4.8	1	6.7
Aboriginal	0	0.0	1	6.7
Filipino	1	4.8	0	0.0
Indian	2	9.5	0	0.0
Missing	0	0.0	1	6.7
Grade Level				
Early Education (Year K-2)	6	28.6	4	26.7
Primary (Year 3–6)	6	28.6	5	33.3
Lower high school (Year 7–9)	1	4.8	0	0.0
Senior high school (Year 10–12)	0	0.0	1	6.7
Multiple grades	8	38.1	5	33.3
**Age in years***M* (*SD*)	38.8 (*SD* = 9.4)		44.8 (*SD* = 10.1)	
**Years Teaching***M* (*SD*)	13.3 (*SD* = 9.3)		18.1 (*SD* = 10.6)	
**Consultants (*n*** **= 14)**	**SAU**		**COMPASS**	
	*n*	%	*n*	%
Gender				
Male	0	0.0	1	12.5
Female	6	100	7	87.5
Ethnicity				
Caucasian	5	83.3	5	62.5
Bengali	0	0.0	1	12.5
Indian	1	16.7	1	12.5
Hispanic	0	0.0	1	12.5
**Age in years***M* (*SD*)	46.0 (*SD* = 8.8)		42.4 (*SD* = 3.9)	
**Years Teaching***M* (*SD*)	19.2 (*SD* = 9.9)		13.9 (*SD* = 7.3)	
**Years Consulting***M* (*SD*)	2.8 (*SD* = 2.4)		3.9 (*SD* = 4.4)	

Note.^1^ All students in the study had a diagnosis of autism and there were no differences observed between the SAU and COMPASS students scores on the SRS-2, *t*(34) = 1.38, *p* = .20

### Measures

#### Baseline and Demographic Measures

**Fig. 1:**
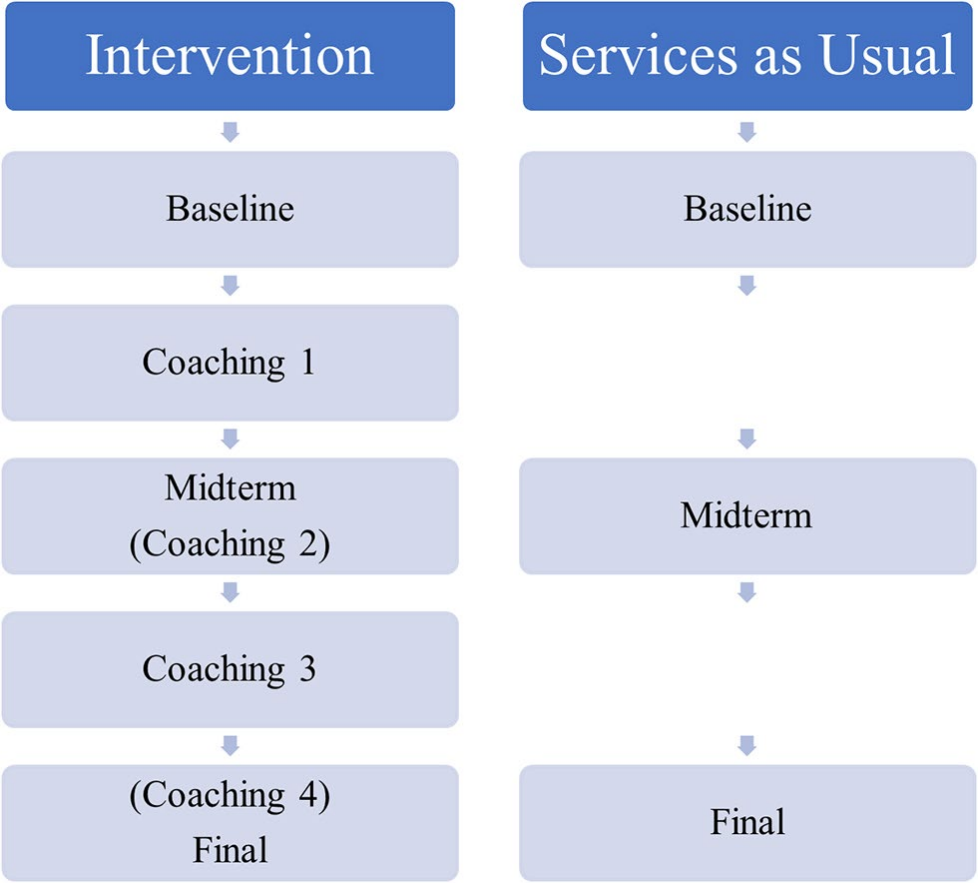
Data was collected across one school year. Baseline data was collected including demographic information and student individualized goals. Midterm and final data included progress on individualized goals, for both groups. The intervention group had extra data collection to coincide with coaching, as consistent with the COMPASS protocol

At the beginning of the study, consultants, caregivers, and teachers were given a baseline demographic survey on Qualtrics (www.qualtrics.com). The caregiver completed the baseline measure on behalf of their child. The questionnaire consisted of demographic variables (e.g., gender, relationship status) and for teachers and consultants, professional details (e.g., number of years working with students with autism, professional training). Sample equivalency was established by gathering information about student demographics and autism diagnoses at baseline. Each student’s caregiver completed an electronic version of the Social Responsiveness Scale Second Edition (SRS-2; Constantino & Gruber, 2012) to give a measure of the severity of social impairment. The background survey took approximately 30 min to complete and all participants completed the survey independently, on their own devices. See Fig. 1 for a data collection schedule.

### Goal Attainment Scaling

**Fig. 2:**
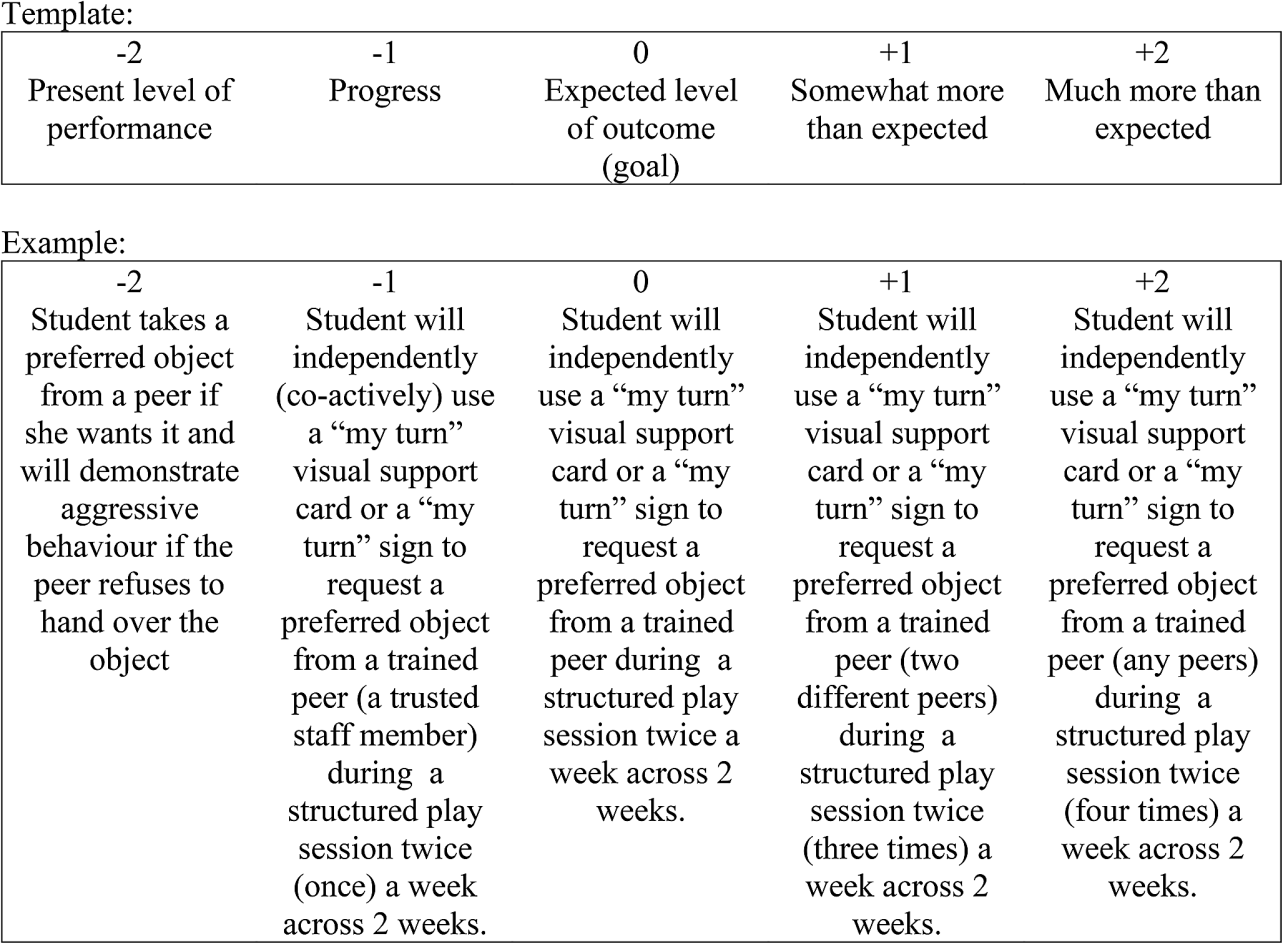
Goal Attainment Scaling Template and Example. *Note* The phrases in parenthesis represent the current expectations for the skills to demonstrate progress at that level. For example, to show “progress” from the present level of performance or to obtain a score of“-1”, the student will need to co-actively demonstrate the skill once a week with a trusted staff member (instead of a peer)

The GAS (Ruble et al., 2012a, b, 2021, 2022) was the primary student outcome measure for this study and supported an evaluation of the student’s performance on their individualized goals across the school year. The tool was designed as an alternative evaluation technique for developing individualized, multivariable, scaled descriptions for outcome measurement and was adapted by COMPASS researchers from a tool commonly used in mental health practice (Kirsuk & Sherman, 1968; Ruble et al., 2022). More recently, GAS has demonstrated progress for autistic individuals in other studies (e.g., Lee et al., 2022). GAS is a way for teachers to write IP goals that include a statement of the present level, the goal, and how the goal looks with some progress and more than expected progress. The GAS template (see Fig. 2) is completed by the team as part of the initial consultation and is used as a progress-monitoring tool throughout the study during the coaching sessions. The scale ranges from "− 2" (which represents the student’s present level of performance) to "+ 2" (which represents much more progress than expected) and requires the teacher and consultant to think through not only the student’s individualized goal but also operationalizing what that goal looks like compared to the present level as well as at the level of exceeding the goal. A score of “0” demonstrates expected student progress toward the actual IP goal from their present level at the beginning of the school year. Goal attainment scaling was shown to be associated with caregiver and teacher self-report ratings of child progress on IP goals as well as to an independent rater (Ruble et al., 2021), and more details on evidence for GAS are summarized by Ruble and colleagues (2012b; 2022). 

Unlike the COMPASS group where GAS goals are written as part of the COMPASS process, for SAU participants, GAS goals were not a part of their standard IP process. Therefore, to make a comparison between the two groups, GAS goals that were observable and measurable were created for all students in the SAU group by a research team member based on their documented IP goals and present-level descriptions. To understand how the quality of GAS goals differed across groups, three quality items (level of difficulty, measurability, equality) were scored for all GAS goals by an independent rater who was blind to group assignment to determine psychometric equivalency. The rater was an educational researcher and associate professor who completed training in GAS and scoring before beginning their blind review. All items were scored using a 3-point Likert-type response scale that was unique to the item (e.g., not at all, somewhat, very). The *level of difficulty* ensures that the goal is the right level of difficulty compared to the student’s present level. *Measurability* confirms the indicators (prompt level, observable skill, criterion for success) are listed for each goal. *Equality* looks at the distance between each GAS level, ensuring equal distance of scaled objectives. To ensure equivalency between the GAS forms of each group, two raters independently coded 20% of the forms using the three quality items. Sample interrater agreement was calculated.

To score the GAS and show progress on student IP goals, all students in the study began at baseline (-2). At each coaching session (COMPASS participants) or IP meeting (SAU participants), progress on the individualized goals was decided jointly by the teacher and consultant. At times, caregiver(s) and students also participated in the coaching sessions. Data in the form of videos, observational field notes, and work samples were used to make this determination. A researcher who was blind to participant groups scored all students’ final GAS goals based on a detailed teacher interview. Before the independent rater began scoring, they were trained based on a set of GAS goals not used in the study. The midterm and final GAS scores were used for data analysis purposes.

### Fidelity of Implementation

Fidelity data were gathered from the intervention group based on the initial consultation using the COMPASS fidelity checklist which outlines the components of a COMPASS consultation. The checklist (see Ruble et al., 2012a; 2023) was adapted from Ruble et al., 2018b and was designed to ensure that consultants followed the COMPASS protocol with fidelity. Participants answered “yes” or “no” to questions about the presence of 25 components of the initial COMPASS meeting or consultation. For example, teachers, consultants, and caregiver(s) were asked to acknowledge if “teaching goals included contributions from each member.” To further confirm the fidelity of consultation implementation, a member of the research team rated fidelity based on direct observations when a researcher was in the consultation or by a review of the video and audio recording of the meeting. The Kuder-Richardson 20 (KR-20) formula was .89 for researchers and .99 for caregiver(s), teachers, and consultants.

Similarly, fidelity to the COMPASS coaching protocol was measured using a 12-item checklist with the components of coaching. Participants responded using a dichotomous “yes” or “not” response format to acknowledge the existence of coaching components. For example, a sample item reads, “we reviewed the most current teaching plan and updated the written plan to reflect current teaching strategies for each objective.” Teachers (*KR20* = .98), consultants (*KR20* = .99), and researchers (*KR20* = .92) completed the items independently.

### Satisfaction

Because this intervention was being introduced across schools where an IP protocol already existed, levels of satisfaction needed to be assessed across participant groups. An 18-item COMPASS satisfaction survey was modified from Ruble and colleagues (2018b) and a 4-point Likert-type response scale ranging from *strongly disagree* to *strongly agree.* A version was used for consultants, teachers, and caregiver(s) in the intervention group. For teachers, a sample item reads, “How likely would you be to recommend the use of COMPASS for teachers of students on the autism spectrum for students with whom they are having difficulty?” Internal consistency (alpha) was .85 for teachers, .82 for consultants, and .86 for caregivers.

### Adherence to Teacher Plans

For our study, we were also interested in how teachers were adhering to their teaching plans. Teaching plans were an element consistent across both the SAU and the intervention group, so understanding if these plans were carried out, could explain student progress at the end of the year. To measure adherence, the consultant answered one question: Did the teacher implement the elements of the teaching plan? Consultants answered using a 5-point Likert-type response scale ranging from *not at all* to *very much*, consistent with Ruble et al. (2018b). For COMPASS participants, this was measured following each coaching session. For SAU participants, this was measured at midterm and final.

### Data Analysis

M*plus* 8.7 (Muthén & Muthén, 2021) facilitated all data analyses, using maximum likelihood estimation with robust standard errors (MLR). Minimal missing data were observed for mid and final GAS scores, restricted only to the SAU group, and due to one or two missing goals (i.e., 3 goals were needed to compute a GAS score). For mid and final GAS scores, 23 of 36 (64%) had complete data: 13 participants had missing data for mid and final GAS scores. Missing data for mid and final GAS scores were addressed by using MLR and estimating the variance for group during the data analysis.

To address the first research hypothesis, we ran a two-level cluster-randomized analysis due to the hierarchical structure of the data, wherein student-teacher dyads are situated within consultants. We ran two models to separately test the hypotheses that group differences would exist both at midterm and final assessment for student GAS outcomes. Mid and final GAS scores were computed based on averaging scores across three goals. If a student was missing one of the goals, the data were treated as missing during the data analysis and was addressed using MLR. Both analyses were performed with an adjusted alpha level from 0.05 to 0.25 using Bonferroni’s correction (0.05/2 = 0.025). The level-1 equation is at the student-teacher dyad level, and used the final GAS scores:

$${FinalGAS}_{ij}={\beta }_{0j}+ {\epsilon }_{ij}$$_,_ [1]

and the level-2 equation is,

$${\beta }_{0j}={\gamma }_{00}+ {\gamma }_{01}GROUP+ {\mu }_{0j}$$, [2]

where FinalGAS_*ij*_ is the final GAS score at the end of the study for student *i* with consultant *j* and β_*0j*_ is the average final GAS score for consultant *j*. For level-1, the error term that is unique to student *i* with consultant *j* is represented by ∑_*ij*_, whereas the error term unique to consultant *j* is represented as *u*_*0j*_ in the level-2 model. The level-2 model accounts for the hierarchy of student-teacher dyads nested within consultants. For the level-2 model, Ⓒ_00_ is the average final GAS score or grand mean, Ⓒ_01_ is the unstandardized effect size on the raw metric of GAS, and GROUP represents the treatment effect (1 = COMPASS, 0 = SAU). The same model was used for midterm GAS scores. The standardized effect size was reported for treatment effects, which used the following equation (Hedges, 2007),

$${\delta }_{T}= \frac{{\gamma }_{01}}{\sqrt{{\sigma }_{W}^{2}+{\sigma }_{B}^{2}}}$$_,_ [3]

where ${\delta }_{T}$ is on the unit of *total SD*, ${\sigma }_{W}^{2}$ represents the within-cluster variance, and ${\sigma }_{B}^{2}$ is the between-cluster variance.

When considering adherence as an outcome, both the ordinal nature of the Likert-type adherence item and hierarchical structure of the data needs to be considered. However, due to the sparseness in the response categories (i.e., zero to few responses observed in the response categories by group), standard errors and points estimates become unstable. Therefore, descriptive statistics (i.e., frequencies, mean, median) were used to compare adherence across groups at both mid and final assessment and adherence across all coaching sessions within the COMPASS group. Finally, when considering fidelity of implementation and satisfaction, descriptive statistics (i.e., means, SD, %) were provided for the COMPASS group. No baseline differences were observed on student variables (see Table 1).

## Results

### Student Outcomes

The first hypothesis examined progress toward students’ IP goals for participants in both groups. Results demonstrate no significant treatment effect for COMPASS on midterm GAS score, γ_01_ = 0.42, *p* = .16, γ_01_ = -1.23, ${\sigma }_{W}^{2}$ = 0.24, ${\sigma }_{B}^{2}$ = 0.20, ${\delta }_{T}$ = 0.63. However, at final assessment, a significant treatment effect exists favoring COMPASS over SAU, γ_01_ = 1.67, *p* < .001, γ_01_ = -0.50, ${\sigma }_{W}^{2}$ = 0.32, ${\sigma }_{B}^{2}$ = 0.002, ${\delta }_{T}$ = 2.94. Additionally, in the COMPASS group at final assessment, 75% of the students met their stated goal at 0 level or higher. In the SAU group, 50% of the students met their stated goal at 0 level or higher. Students in the COMPASS group also received higher ratings of goal quality, based on three indicators compared to the SAU group.

### Adherence

Teacher adherence across coaching sessions for both groups is reported in Table 2. As observed in Table 2, both mid (Coaching session 2) and final (Coaching session 4) mean and median teacher adherence levels are descriptively higher for the COMPASS group relative to the SAU group. Additionally, as shown in Table 2, mean and median teacher adherence levels descriptively increase across coaching sessions within the COMPASS group.

**Table 2:** Descriptive statistics for teacher adherence by coaching session and group

Coaching session	COMPASS					**SAU**				
	*Min*	*Max*	*M*	*Med*	*SD*	*Min*	*Max*	*M*	*Med*	*SD*
1	1	3	2.50	3.00	0.73					
2 - Midterm	1	5	2.88	3.00	0.89	1	3	1.85	2.00	0.81
3	1	5	3.31	3.00	1.25					
4 – Final	2	5	4.31	4.50	0.87	1	5	3.15	3.00	1.23

*Note* Minimum and maximum possible adherence scores are 0 (Strongly Disagree) and 5 (Strongly Agree), respectively. Adherence data were only collected at Coaching sessions 2 and 4 for SAU

### Fidelity and Satisfaction

According to researcher fidelity scores, consultants demonstrated 21.07 components out of 25 COMPASS components. That is, researchers observed 84.4% of the consultation components of COMPASS during the initial consultation component of COMPASS. For coaching sessions, researcher fidelity resulted in a mean of 14.5 (*SD* = 1.16). That is, 90.6% of the coaching components of COMPASS were observed across coaching sessions conducted by consultants.

### Satisfaction

Teachers in the COMPASS group reported a mean satisfaction (on a 1 to 5 scale) score of 3.1 (*SD* = 0.43) on 18 satisfaction-related questions, and consultants reported a mean satisfaction score of 2.8 (*SD* = 0.42). Both participant groups were most satisfied with (a) the GAS and the way they were taught to measure goals (*M* = 3.67, *SD* = 0.49), (b) the assessment form used by caregiver(s) and teachers before the first IP meeting (*M* = 3.40, *SD* = 0.52), and (c) the quality of the goals identified by the process for each student (*M* = 3.40, *SD* = 0.51). They were least satisfied with burdens related to time and resources (*M* = 2.33, *SD* = 0.82). Caregiver(s) in the study reported a mean satisfaction (on a 1 to 5 scale) score of 3.2, *SD* = 0.60 and were most satisfied with how the process allowed them to know about their child’s progress (*M* = 3.43, *SD* = 0.78) and what strategies were being used to teach their child (*M* = 3.43, *SD* = 0.79).

## Discussion

The primary aim of the study was to understand if COMPASS would improve student individualized goal outcomes within an autism-specific context in Australia. Students in the intervention group made more progress on their individualized goals, which replicates work by Ruble et al. (2010; 2013; 2018a), and is the first study to replicate this intervention. Additionally, our study took an important step to determine the effectiveness of COMPASS independent of the COMPASS developers and delivered with satisfaction and fidelity by educators, and not researchers. The success of this intervention in demonstrating progress towards IP goals for students on the autism spectrum highlights the need for a standardized intervention that supports teachers in this critical practice.

Teaching students on the autism spectrum requires expertise, knowledge, and proficiency and the research is still emerging to guide teachers in the best practices that will lead to student progress and achievement. Teachers of autistic students often report high levels of stress and burnout (Boujut et al., 2017; Love et al., 2020) and there have been few studies focused on ways to better support these teachers in supporting the learning of autistic students. As pointed out by Ruble and colleagues (2013), interventions that are designed to address outcomes across core individualized areas and are not specific to one skill are crucial in seeing improvements in autism teaching practices and standards. While evidence-based interventions for explicit educational areas such as reading, writing, speaking, or social skills may be useful, an intervention like COMPASS may provide more support to teachers and caregivers because it addresses the collaborative problem-solving process teachers engage in daily across all teaching practices. It addresses the *process* of teaching instead of focusing on just one area of improvement for their students or one specific practice such as the evidence-based practices identified by Wong and colleagues (2015). The success demonstrated in our study and previous COMPASS work further highlights the need for rigorous research demonstrating the impact of a process-related intervention. In Australia, the Disability Standards for Education (2006) require consultation with the student and/or their parents or carers regarding adaptations to the curriculum of a course or program for students on the autism spectrum, but no process is suggested or mandated (Dickenson, 2014). Therefore, COMPASS may provide a research-validated process for Australian teachers to refer to.

Uniquely, our study found significant differences in final GAS scores only with no. evidence of differences between groups at midterm. This could be due to the timing of the COVID-19 lockdowns for the cohort within this study. All students, at midterm, were completing a period of home learning. By the time final scores were collected, students had returned to school and face-to-face learning. During this period between midterm and when final scores were collected, students in the COMPASS group made more progress than those in the SAU group. Ruble and colleagues (2013) have previously demonstrated efficacy for a virtual or web-based version of COMPASS however, it is possible that for our context, face-to-face learning (i.e., not virtual home learning) was more effective. Future work that addresses the question of virtual COMPASS delivery for students on the autism spectrum in Australia and which seeks to replicate Ruble and colleagues (2013) work is justified as well as replication of this study outside of COVID-19. For Australia, the delivery of COMPASS as a virtual intervention would be of interest to caregivers and families, considering the challenges faced by many families in rural settings in receiving support and accessing appropriate educational systems and services for their students on the autism spectrum (Ault et al., 2021).

This study was objectively different from past work by Ruble and colleagues (2010, 2013, 2018a). Specifically, the study was conducted in an Australian context, with community consultants, utilizing modifications specific to this context. In our study, community consultants were senior educators who held consulting and coaching roles at their schools. Additionally, the study was conducted across the age range, while previous work has focused on one specific age group. However, despite these differences, the results of our study are comparable to previous COMPASS research. For example, Ruble and colleagues (2018a), who focused on transition aged-youth, found that 67% of students who participated in COMPASS met their individualized goal, compared to just 18% in the SAU group. In our study, a similar proportion of students in the COMPASS group met their individualized goal (70%) however a larger proportion of the SAU group (50%) also met their goal. The success of the SAU group in our study may be due to the existing protocol in place for individualized planning within our schools. It could be expected that teachers at an autism-specific school, like those in our study, would have a greater understanding of research-based practices for autistic students, which many not have been the case in previous work by Ruble and colleagues.

Throughout the study, the research team considered sustainability of the COMPASS intervention, beyond the research project. The team discussed how COMPASS can be delivered school-wide in a school where all students are on the autism spectrum. Some of the challenges discussed included how to manage the time resource and sustainability of the consultation model. Although meaningful and valued by participants in our study, the initial consultation may not be manageable across the whole student body due to a lack of resources and time. Additionally, due to time and resource constraints, an internal consultant could not closely mediate all consultation and coaching meetings if all students participated in COMPASS. One suggestion was the option for teachers to work in collaboration in professional learning communities to keep each other accountable for their student’s individualized plans instead of requiring consultant accountability. Additionally, we discussed how COMPASS could be used for students who may require more Tier 3 intervention instead of for all students at the school. These suggestions may make COMPASS more sustainable; however, we recommend future research is designed to consider the feasibility and sustainability of COMPASS in schools where all students are on the autism spectrum.

### Limitations

There are several limitations worth discussing in our study. First, one challenge faced during data analyses was the missing data observed for some students in the SAU group who had fewer than three goals. COMPASS naturally assigns a communication, learning, and social goal to each student. Yet, students in the SAU group did not have a standardized set number of goals equal to those in the COMPASS group. To address this limitation, we used MLR and computed mid and final GAS scores based on complete data. Although a limitation, the results are encouraging given that the direction and size of effects for GAS mimicked those observed by Ruble and colleagues (2013; 2018a). Future studies should overcome this limitation by requiring both COMPASS and SAU groups to use the same number and type of goals. Additionally, this study, along with previous work in COMPASS, used GAS as the primary outcome to track the growth on students’ individualized goals. Although there is strong evidence for GAS outside of COMPASS (e.g., Lee et al., 2022) and work has been done to establish GAS as a reliable and valid measure (e.g., Ruble et al., 2012a, b, 2021), it is not known if the success of COMPASS is reliant on the use of GAS. Future iterations of COMPASS could alleviate this limitation by replicating COMPASS with a different outcome measure. Of note, the study was conducted during the 2021 school year, which included numerous COVID-19-related disruptions, so more research is warranted in a more typical school year as COMPASS components are rolled out.

Finally, an important consideration for this study compared to previous COMPASS work is the setting of the study. As this study was conducted in an autism-specific school, the generalizability to other settings (e.g., mainstream settings or schools designed for students of all disabilities) is unknown. Research is needed to compare the results of this study to other settings in Australia and to understand how the process of individualized goal setting might vary from one context to another. All Aspect staff members are trained in neuroaffirming goal setting and engage in regular conversations about the importance of neurodiversity. These may have influenced the success of COMPASS in our setting, but future research is warranted to understand this context compared to another.

## Conclusion

The COMPASS intervention demonstrated success for the participants in our study, and it is recommended that additional research is conducted with diverse samples and across various school contexts. The project replicated an intervention that helps to improve the quality of goals and progress for school-age students on the spectrum, a challenge that has been acknowledged in practice and research for years. The intervention warrants additional research and knowledge sharing to continue improving educational opportunities for students on the autism spectrum and to ensure successful scalability.

## Author’s Note

The authors are aware of different preferences and reasoning regarding the use of person-first (e.g. person with autism) versus identity-first (e.g. autistic person) language. The authors have chosen to write this paper using identity-first language, as this is the preference of most autistic people, including those on the research team (Bury et al., 2020; Taboas et al., 2022).
